# Laveran, le médecin militaire

**DOI:** 10.48327/mtsi.v3i1.2023.324

**Published:** 2023-02-24

**Authors:** René Migliani

**Affiliations:** SFMTSI Société francophone de médecine tropicale et santé internationale (ancienne SPE), Hôpital Pitié-Salpêtrière, Pavillon Laveran, 47-83 Boulevard de l'Hôpital, 75651 Paris cedex 13, France; * Actes du Colloque – Centenaire de la mort d'Alphonse Laveran. 24 novembre 2022, Paris / Proceedings of the Conference – Centenary of the death of Alphonse Laveran. 24 November 2022, Paris

**Keywords:** Alphonse Laveran, Médecin, Militaire, Paludisme, Hématozoaire, Protozoologie, Prix Nobel, Alphonse Laveran, Military doctor, Malaria, Haematozoa, Protozoology, Nobel Prize

## Abstract

Fils de Louis-Théodore Laveran titulaire de la Chaire des maladies et épidémies aux armées du Val-de-Grâce et petit-fils par sa mère d'un commandant d'artillerie, Alphonse, né à Paris le 18 juin 1845, suit les traces paternelles en intégrant à 18 ans l’École impériale de santé militaire de Strasbourg.

Après sa thèse, il participe en 1870 à la guerre contre la Prusse. Il est fait prisonnier à Metz. Il prépare ensuite le concours de professeur qu'il réussit en 1874. Il est agrégé à la Chaire du Val-de-Grâce que son père avait créée. Il rejoint ensuite l'Algérie. C'est à l'hôpital militaire de Constantine, le 6 novembre 1880, qu'il met en évidence de façon indiscutable dans le sang d'un militaire du train des équipages, l'hématozoaire responsable du paludisme.

En 1884, il est nommé titulaire de la Chaire d'hygiène militaire et de médecine légale du Val-de-Grâce. En fin de professorat en 1894, il essuie un refus d'affectation sur Paris pour y poursuivre ses recherches. Non consulté en outre pour la préparation de l'expédition de Madagascar, qui tourne en 1895 au désastre sanitaire, il prend prématurément sa retraite en 1897. Accueilli par Émile Duclaux et Émile Roux à l'Institut Pasteur à Paris, il poursuit ses recherches principalement sur les protozoaires comme agents de maladies humaines et animales jusqu’à sa mort. L'ensemble de ses travaux de protozoologie médicale lui vaudra d'obtenir le prix Nobel de physiologie ou médecine en 1907. Pendant la Grande Guerre, fort de son expérience, il met en garde en janvier 1916 le ministre de la Guerre, sur le risque palustre encouru par l'armée d'Orient dans le delta du fleuve Vardar à Salonique. Le printemps lui donnera raison.

Illustre médecin militaire et scientifique de renommée internationale, Laveran s’éteint le 18 mai 1922 à Paris.

## Abstract (en)

Son of Louis-Theodore Laveran, holder of the Chair of Diseases and Epidemics in the Armies at the Val-de-Grâce and grandson of an artillery commander through his mother, Alphonse, born in Paris on June 18, 1845, follows in his father's footsteps by entering the Imperial School of Military Health in Strasbourg at the age of 18.

After his thesis, he participated in 1870 in the war against Prussia. He was taken prisoner in Metz. He then prepared for the competitive examination to become a professor, which he passed in 1874. He was appointed to the Chair of the Val-de-Grâce, which his father had created. He then went to Algeria. It was at the military hospital in Constantine on November 6, 1880 that he indisputably discovered the haematozoa responsible for malaria in the blood of a soldier in the crew train.

In 1884, he was appointed to the Chair of Military Hygiene and Legal Medicine at Val-de-Grâce. At the end of his professorship in 1894, after being refused a posting to Paris to continue his research and not being consulted for the preparation of the Madagascar expedition, which turned into a health disaster in 1895, he retired prematurely in 1897. Hosted by Émile Duclaux and Émile Roux at the Pasteur Institute in Paris, he continued his research mainly on protozoa as agents of human and animal diseases until his death. His work in medical protozoology earned him the Nobel Prize in Physiology or Medicine in 1907. During the Great War, with the benefit of his experience, he warned the Minister of War in January 1916 about the risk of malaria incurred by the army of the East in the delta of the Vardar River in Salonika. The spring would prove him right.

An illustrious military doctor and scientist of international renown, Laveran died on May 18, 1922 in Paris.

## Introduction

À défaut du Panthéon national, Alphonse Laveran mérite sans conteste de figurer en 2022 dans le panthéon personnel des médecins à plus d'un titre, dont le plus prestigieux est le prix Nobel de médecine qui couronna pour la première fois un savant français en 1907. Voici à l'occasion de la commémoration du centenaire de son décès, ce que fut sa vie de médecin militaire, une synthèse réalisée à partir de ses livres et publications et des témoignages de ceux qui le côtoyèrent et de ceux qui commémorèrent son souvenir jusqu’à nos jours.

## Jeunesse D'alphonse Laveran

### Racines familiales

Louis, le grand-père d'Alphonse, né à Montesquieu-de-Volvestre près de Carbonne, à 40 km au sud de Toulouse, était chirurgien militaire puis dentiste installé en Flandre maritime où est né en 1812 à Dunkerque, Louis-Théodore père d'Alphonse, qui deviendra un brillant médecin militaire [4]. Le parcours paternel est en effet éloquent: professeur, titulaire de la Chaire des maladies et épidémies aux armées qu'il créa au sein de l’École du Valde-Grâce. Il terminera sa carrière comme directeur du Val-de-Grâce en 1872, en « chef aimé et respecté et en maître écouté » [4, 14].

Le grand-père maternel d'Alphonse était artilleur et sa femme Marie Marguerite, née Lallemand à Metz, était la sœur de deux illustres généraux d'Empire: François Antoine, cavalier, Pair de France et Henri Dominique, artilleur, blessé à Waterloo, auteur d'un traité d'artillerie [13, 31].

On devine aisément que la forte image paternelle et les prestigieuses racines familiales militaires ont pu influencer les choix futurs du jeune Alphonse.

### Naissance, enfance et scolarité

Alphonse Laveran est né le 18 juin 1845 à Paris, son père étant à ce moment-là professeur en second à l’École du Val-de-Grâce (EVDG). Suivant ensuite son père lors de ses multiples mutations, Alphonse découvrira à 2 ans la garnison de Metz, puis en 1851 la ville de Blida en Algérie, à 40 kilomètres d'Alger au pied de l'Atlas, dont il garda un souvenir enchanteur, avant de revenir à Paris en 1856 à l’âge de 11 ans [18, 42]. À son retour à Paris, où son père est muté à l'EVDG, il est scolarisé au collège Sainte-Barbe, sur la Montagne Sainte-Geneviève dans le 5^e^ arrondissement, puis au lycée Louis-le-Grand où il obtiendra son baccalauréat à 18 ans [42].

## Formation De Médecin Militaire

Son baccalauréat en poche, Laveran suit les traces de son père qui fut sans doute son conseiller et son meilleur guide [14]. Il est admis le 29 octobre 1863, quatrième sur cent, au concours d'entrée à l’École impériale du service de santé militaire de Strasbourg avec l'appréciation « extrêmement satisfait » (note n° 1). Charles-Emmanuel Sédillot, brillant chirurgien, à qui l'on devra en 1878 l'invention du mot « microbe », la dirigeait depuis sa création en 1856 [6, 17]. Laveran devient un « carabin rouge » [40]. Il suit un enseignement théorique à la Faculté de médecine et une formation pratique aux Hospices civils et à l'hôpital militaire de Strasbourg [40], ce qui est encore globalement la formation des médecins des armées en 2022. Lors de son cursus, sa situation d’étudiant est excellente et sans échec avec des notes variant de « bien satisfait » à « très distingué » [17].

En 1865, il est reçu au concours de l'externat puis en 1866 à celui de l'internat de l'hôpital civil de Strasbourg. Il soutient brillamment, avec la « mention honorable », sa thèse de doctorat le 29 novembre 1867 sur un sujet de recherche intitulé « Recherches expérimentales sur la régénération des nerfs » chez l'animal « objectivée par l'examen histologique et prouvée par la récupération fonctionnelle » [17, 19, 42]. De cette période de formation médicale à l’École impériale et à la Faculté civile, Laveran dira qu'il y a « appris la rigueur », ce dont la qualité de sa thèse en est la preuve éclatante [1, 17]. Laveran est pendant ses études l’élève de Charles Morel, membre de son jury de thèse, professeur de la Faculté de médecine de Strasbourg, pionnier de l'histologie, qui va l'initier à la pratique du microscope et à l'examen des lésions anatomo-pathologiques [39]. En 1860, Morel a publié un précis d'histologie humaine de référence [35].

## Parcours Au Sein Du Service De Santé Militaire

### Première affectation à Paris

Alphonse Laveran sort au deuxième rang du stage d'application du Val-de-Grâce, ce qui lui vaut le privilège de pouvoir choisir un hôpital parisien. Il est ainsi affecté à l'hôpital militaire Saint-Martin, rue des Récollets à Paris en octobre 1868 avec le grade de médecin aide-major de 2^e^ classe [6]. La première appréciation sur sa manière de servir est élogieuse et prometteuse. On en retiendra la conclusion: « Sujet d'avenir » [14].

### Guerre franco-prussienne de 1870

Le 19 juillet 1870, Napoléon III déclare la guerre à la Prusse. Laveran, âgé de 25 ans, est affecté dans l'Armée du Rhin à l'ambulance de la 3^e^ division du 3^e^ corps d'armée (CA) commandé par le Maréchal François Bazaine à Metz [14]. Le 18 août, après la défaite de Saint-Privat (victoire de Gravelotte pour les Prussiens), commence le blocus de Metz où sont repliées les troupes du 3^e^ corps. Laveran est affecté à l'ambulance de l’île de Chambières, réputée « la plus mauvaise » [36]. Grâce à des ballons qui passent du courrier au-dessus des lignes prussiennes, il rassure ses parents par des messages simples et laconiques: « Je me porte bien », écrits sur du papier pelure [36].

Napoléon III capitule à Sedan le 2 septembre, puis Bazaine à Metz le 27 octobre. Début novembre, Laveran quitte Metz avec son ordonnance muni d'un sauf-conduit sanitaire et rejoint l'hôpital militaire de Lille dont son père est le directeur [6, 42]. Il sert jusqu'au 22 mars 1871 et est promu médecin aide-major de 1^re^ classe [6, 14]. Plus tard il s'ouvrira en évoquant « qu'il était de la malheureuse armée de Metz » [32].

### Commune de Paris

La guerre se poursuit jusqu’à l'Armistice le 28 janvier 1871, après 4 mois de siège de la capitale. Le 18 mars une insurrection éclate à Paris. De retour le 22 mars à l'hôpital Saint-Martin, Laveran s'active à son poste, notamment pendant la « Semaine sanglante » de la Commune qui va durer jusqu'au 28 mai avec des barricades rue des Récollets [42].

La III^e^ République est proclamée en septembre 1871. À la fin de son affectation en 1872, son médecin-chef donne une fois de plus une très bonne appréciation sur sa manière de servir, qui éclaire sur ses qualités d'organisateur et d'enseignant. Il écrit: « A. Laveran a servi sous mes ordres à Saint-Martin, souvent comme chef de service et toujours comme le moniteur de ses camarades, assurant tous les détails imprévus d'un service difficile, surtout pendant la Commune [14]. »

### Régiment de hussards à Pontivy en Bretagne

Après un détachement à l'hôpital thermal militaire de Vichy, Laveran est affecté au 10^e^ régiment de hussards à Pontivy en mars 1873. Il est promu au grade de médecin-major de 2^e^ classe et prépare le concours d'agrégation de médecine [6]. Cette année-là son chef de corps, le Colonel de Saint-Jean, donnait une nouvelle fois une appréciation soulignant sa valeur et son potentiel par la formule visionnaire « Médecin d'avenir » [14].

### Agrégation de médecine au Val-de-Grâce

Ayant réussi le concours, Laveran est agrégé en 1874 à la Chaire des maladies et épidémies aux armées, dont le titulaire est Léon Colin qui avait succédé à son père Louis-Théodore. En plus de ses activités d'enseignant, il se perfectionne, comme à Strasbourg, en anatomo-pathologie dans les laboratoires du Val-de-Grâce et dans celui du Professeur Louis Ranvier au Collège de France. Ranvier est un neurohistologiste célèbre au xix^e^ siècle, dont le nom reste associé à la découverte sur les axones des neurones des « nœuds de Ranvier ». Il fut un chef de file dans la microscopie et dans le renouveau de l'anatomie générale française [2]. Il étudia entre autres « la dégénérescence des nerfs après leur section », ce qui explique peut-être le choix de Laveran pour ce laboratoire. Enfin en 1875, Laveran rédige un *Traité des maladies et épidémies des armées* [20].

### Division de Constantine en Algérie

À la fin de son agrégation à 33 ans, Laveran est désigné pour servir dans les hôpitaux de la division de Constantine en Algérie. Le paludisme y est toujours fréquent et grave, en particulier dans les zones côtières. Un premier médecin militaire, François Clément Maillot avait démontré, en 1834 et 1835 à l'hôpital militaire de Bône, l'efficacité du traitement immédiat des fiévreux paludéens avec de fortes doses de sulfate de quinine, contrairement à l'enseignement de Broussais au Val-de-Grâce. Il fallut plusieurs années à Maillot pour rallier le corps médical à ses idées qui seront définitivement reconnues lors du congrès scientifique organisé à Alger en 1881 [3, 33].

Laveran, qui n'ignore rien des travaux de Maillot, est d'abord affecté à l'Hôpital militaire de Bône en 1878 puis à celui de Biskra en 1879 [14].

À Bône, Laveran accueille un grand nombre de paludéens. Il réalise ses premières recherches en pratiquant des autopsies sur des militaires morts de fièvre pernicieuse qui confirmèrent que la « seule lésion caractéristique de l'impaludisme [ainsi que l'on dénomme le paludisme à cette époque] consistait dans la présence d’éléments pigmentés dans le sang ». Pour comprendre l'origine de ces éléments, il décide d’étudier « de ses yeux myopes au travers de son microscope » de marque Verick, les éléments pigmentés dans le sang frais de sujets infectés, entre lame et lamelle sans coloration. Il découvre ainsi des « corps sphériques pigmentés avec mouvements amiboïdes », qu'il dénomme « Corps n° 1 ». Il indique dans son cahier de notes: « Je supposai dès lors qu'il s'agissait de parasites » [14,17,27,28,32,38]. Ceci est une nouveauté à une époque où aucun parasite n'a été isolé jusque-là dans le sang de sujets malades [14]. À Biskra, il étudie la leishmaniose cutanée due à *Leishmania tropica* responsable du « clou de Biskra ». Il est nommé médecin-major de 1^re^ classe le 26 avril. L'appréciation de son médecin-chef est la suivante: « Médecin très distingué, travailleur infatigable, manque de liant avec ses inférieurs. » Son abord froid et distant qui déconcerte et son caractère réservé s'affirment, ne rendant pas son commerce toujours facile [14].

### Découverte de l'agent du paludisme

Laveran va poursuivre ses recherches sur « l'impaludisme » à l'hôpital militaire n° 303 de Constantine, surnommé la « Kasbah », à partir de 1880, où comme à Bône les malades impaludés sont nombreux [18, 29].

À force de persévérance, le 6 novembre 1880, Laveran observe dans le sang frais prélevé au moment d'un accès fébrile et avant traitement, chez un militaire du Train des équipages cantonné au Bardo en bordure des marécages de l'oued Rummel en contrebas de la ville de Constantine, d'une part des « Corps n° 1 » et d'autre part des « Corps n° 2 avec des filaments périphériques mobiles » non observés jusque-là et qui correspondent à un phénomène accidentel à l'air libre dans le sang frais: l'exflagellation des gamétocytes mâles de l'hématozoaire. Ce phénomène sera démontré quelques années plus tard [12, 28, 32]. Laveran note dans son cahier: « Plus de doute sur la nature parasitaire des éléments » observés *in vivo* [14, 37]. Il rédige rapidement un court manuscrit intitulé « Note sur un nouveau parasite trouvé dans le sang de plusieurs malades atteints de fièvre palustre » qu'il envoie à Léon Colin, son ancien titulaire de Chaire, sous-directeur du Val-de-Grâce et nouveau membre de l'Académie de médecine pour présenter cette découverte à celle-ci [10, 42].

Colin qui n'est pas convaincu par la démonstration, partisan lui-même de l'origine tellurique du paludisme, présentera cette découverte le 23 novembre avec « de fortes réserves » [5, 9, 36]. Au cours des semaines suivantes, Laveran adressera d'autres notes complémentaires et il éditera finalement en 1881 un document de plus de 100 pages intitulé *Nature parasitaire des accidents de l'impaludisme. Description d'un nouveau parasite trouvé dans le sang des malades atteints de fièvre palustre* [21].

### Titulaire de la Chaire d'hygiène et de médecine légale du Val-de-Grâce

De retour en métropole en juin 1883, Laveran est affecté à l'hôpital militaire du Gros-Caillou, rue Saint-Dominique à Paris [42]. L'année suivante il est nommé titulaire de la Chaire d'hygiène militaire et de médecine légale du Val-de-Grâce et le restera pendant 10 ans [6]. Il enseigne l'hygiène appliquée aux armées, associant enseignement théorique et pratique, et poursuit l'organisation d'un musée d'hygiène [25]. Il termine la rédaction de son *Traité des fièvres palustres avec la description des microbes du paludisme*, publié cette même année [22, 42]. Dans ce traité, il évoque la probable transmission du paludisme par les moustiques. Il écrit en effet « Les moustiques jouent-ils un rôle dans la pathogénie du paludisme comme dans celle de la filariose? », par analogie avec les travaux du médecin britannique Patrick Manson en Chine du sud en 1878 [8]. Il précise: « La chose n'est pas impossible, il est à noter que les moustiques abondent dans toutes les localités palustres [22]. »

À 40 ans, il est nommé médecin-principal de 2^e^ classe. Dans ses notations, il est indiqué que « les choses de l'administration ne l'intéressent que médiocrement ». Il épouse en octobre 1885 Sophie-Marie Pidancet, 27 ans, à Montoy-Flanville, le village natal de celle-ci en Lorraine occupée [36]. Elle l'aidera tout au long de sa carrière de savant et lui survivra. Ils n'auront pas d'enfants. Peu de temps après, Laveran est fait chevalier de la Légion d'honneur à l'ancienneté le 24 juin 1886, décoration qu'on lui refusa après la guerre de 1870 [6].

### Début de la reconnaissance

Laveran rapporte en 1889 dans les *Archives de médecine expérimentale* que Charles Bouchard, professeur de pathologie et thérapeutique générale et membre titulaire de l'Académie des sciences, après l'observation de flagelles dans le sang frais d'un sujet impaludé, lui apporte son soutien lors de la séance du 21 janvier 1889 de cette académie. Il déclare au cours de cette séance: « Une note récente de M. Laveran me conduit à signaler l'importance d'une découverte qui remonte à dix années et qui, contestée pendant longtemps, me paraît aujourd'hui inattaquable [24]. »

C'est le début de la reconnaissance pour Laveran. Le 31 décembre 1889, sur un rapport de Bouchard, le Prix Bréant lui est décerné à l'unanimité du jury de l'Académie des sciences pour sa découverte de l'hématozoaire [6, 24, 32]. Léon Colin, maintenant convaincu par sa découverte – le paludisme est provoqué par un parasite – écrit: « Digne héritier d'un nom illustre dans la médecine militaire, A. Laveran a fait sur la nature des fièvres intermittentes une découverte qui est un honneur pour le Corps de Santé [14]. » Plusieurs chercheurs accumulent des preuves sur l'origine parasitaire de la découverte de Laveran. Celui-ci avait ainsi présenté, lors de la séance de juillet 1885 de la Société médicale des hôpitaux, les résultats d'une étude importante des Italiens Etore Marchiafava et Angello Celli qui démontrait la transmission de l'hématozoaire de sujets infectés à des sujets indemnes [23]. Ce sont ces deux chercheurs qui proposent la même année de dénommer « *Plasmodium »*, l'hématozoaire [42].

Laveran poursuit sa progression militaire en étant nommé médecin-principal de 1^re^ classe en 1891, ce qui correspond au grade actuel de médecin en chef ou colonel dans la hiérarchie militaire. Il ne sait pas que ce sera son dernier grade [42].

En 1892, le jour des 70 ans de Louis Pasteur, Laveran montre à celui-ci, pour le convaincre de sa découverte, une préparation au microscope avec « au beau milieu du champ un magnifique corps flagellé [qui agite] ses prolongements ». Pasteur est convaincu et admiratif [32].

Puis Laveran est élu à l'Académie de médecine en 1893 à l’âge de 48 ans [41]. Il en devient un membre très actif jusqu’à la fin de sa vie. D'ailleurs en 1920 à 75 ans, alors qu'il est président « de cette compagnie », il en organisera la célébration du centenaire [44].

### Expédition de Madagascar de 1895

De façon surprenante, Laveran n'est pas sollicité à partir de 1893 pour la préparation de l'expédition de Madagascar qui « serait la guerre des médecins » comme le précisera en 1922 dans la nécrologie de Laveran, le médecin inspecteur général Louis Vaillard [14, 45]. Cette expédition se solde en 1895 par un « désastre sanitaire » avec 8 000 à 8 500 décès dont 72% par paludisme, soit un quart de l'effectif expéditionnaire, contre une quinzaine de tués et quelques dizaines de blessés au combat [30]. Laveran fera un rapport à l'Académie de médecine où il n'hésite pas à dénoncer le coût en vies humaines [28]. Louis Vaillard résume parfaitement, avec un certain lyrisme, le sentiment sans doute dominant, par ces mots: « Les tombes creusées par le paludisme de Majunga à Tananarive ont crié ce qu'il en coûta à l'armée de ne pas mettre à sa place l'homme qui convenait à la situation [45]. »

### Fin de carrière militaire de Laveran

À l'issue de son Professorat en 1894, Laveran sollicite sans succès sa nomination à la Chefferie de l'hôpital militaire de Vincennes afin de pouvoir assister aux séances de l'Académie de médecine. Sa hiérarchie le mute au poste de médecin chef de la Place et de l'hôpital militaire de Lille, qu'il connaissait depuis la guerre de 1870. Laveran est ensuite muté au poste de directeur du service de santé du 11^e^ Corps d'armée (CA) à Nantes en 1895, droite ligne pour un avancement vers le grade de médecin-inspecteur à l'instar de son père [14, 42].

Obstiné, Laveran sollicite de nouveau un autre poste, celui de directeur de santé du 5^e^ CA à Orléans qui doit se libérer en 1896, toujours pour se rapprocher de Paris [42]. Le 5 octobre la réponse du général Billot, ministre des armées, celui de l'affaire Dreyfus, est cinglante. C'est un nouveau refus. Le ministre ajoute, à destination du général commandant le 11^e^ CA, de « rappeler au médecin principal Laveran qu'il n'est pas le seul médecin militaire à faire partie de l'Académie » [6]. Laveran préfère alors adresser sa démission. Celle-ci étant acceptée, il est mis en retraite anticipée le 17 février 1897 à l’âge de 51 ans [6].

## Deuxième Carrière À L'institut Pasteur À Paris

### Recherches sur les protozoaires

Laveran, ayant pour but principal de poursuivre ses recherches, est reçu en 1897 par Émile Duclaux et Émile Roux, qui a été son élève au Val-de-Grâce lorsqu'il était agrégé et qu'il initia à la microscopie avant son exclusion pour « bricolage biologique » [15]. Ils lui ouvrent les portes de l'Institut Pasteur où il poursuivra ses travaux sur les protozoaires. Louis Pasteur était mort deux ans plus tôt. La collaboration de cet « aristocrate de la recherche », comme le décrira André Dodin lors du centenaire de la découverte de l'hématozoaire du paludisme à Strasbourg, est toujours désintéressée et le restera jusqu’à son décès [6, 15]. Durant cette période, il continue également à faire bénéficier de son expérience le service de santé militaire, tout particulièrement les médecins des Troupes de marine qui luttent contre les grandes endémies parasitaires [6, 13].

### Traité du paludisme

Laveran termine la rédaction de son *Traité du paludisme*, son exposé magistral, qui paraît en 1898, un an après que Ronald Ross, médecin militaire britannique de l'Indian Medical Service, a apporté la preuve à Calcutta, le dimanche 20 août 1897 (« Mosquito day »), que la transmission du paludisme des oiseaux se faisait par le moustique anophèle femelle [7,11,16,26,29]. Ross recevra en 1902 le 2^e^ Nobel de médecine pour cette découverte. Lors de son banquet, le 10 décembre à Stockholm, Ross rendra un fervent hommage à Laveran au début de son discours par ces mots: « Je commencerai par le grand nom de Laveran, qui a découvert il y a plus de vingt ans la cause du paludisme et a créé une nouvelle branche de la science. Laveran, ce véritable homme de science qui m'a honoré en me permettant de l'appeler mon maître [« my master » dans le texte original] [39]. » Dans ses *Mémoires*, Ross rapporte que durant son séjour en Inde, il échangea plusieurs lettres avec Laveran entre février 1896 et janvier 1899, dont deux en avril et juillet 1898 concernant sa propre découverte [5]. Ces lettres font partie de plusieurs documents confiés par Laveran à Marie Phisalix qu'il charge de l'analyse de son œuvre scientifique [38].

La vie de Laveran se partage dorénavant à Paris entre l'Institut Pasteur, la Société médicale des hôpitaux, l'Académie de médecine et l'Académie des sciences où il est élu comme membre titulaire de la section de médecine et chirurgie en mai 1901, « après une lutte particulièrement ardente » tant la concurrence était grande [6, 32]. La même année, Laveran est promu officier de la Légion d'honneur au titre des réserves. Il est rayé des cadres militaires à la limite d’âge de son grade, le 20 mai 1903 [6].

### Prix Nobel de physiologie ou médecine

En 1907, plus d'un quart de siècle après sa découverte de l'hématozoaire du paludisme, Laveran devient le premier lauréat français du prix Nobel de physiologie ou médecine pour « ses travaux sur l'importance des protozoaires comme agents de maladies », couronnant sa découverte majeure de 1880 et l'ensemble de ses recherches sur les protozoaires pathogènes pour l'homme et l'animal qu'il réalisa à l'Institut Pasteur. Le discours du jury prévu pour la remise du prix précise que l'on pouvait « apprécier à leur juste valeur la perspicacité et l’œil vif de Laveran ». Ce discours ne sera pas prononcé après l'annulation de la cérémonie en raison du décès du roi Oscar II le 8 décembre [43]. Pour la petite histoire, Laveran est lauréat la même année que l’écrivain britannique Rudyard Kipling, prix Nobel de Littérature. Laveran consacrera une partie de son prix à l'aménagement d'un laboratoire de parasitologie à l'Institut Pasteur [32].

En 1912, Laveran reçoit du médecin inspecteur Troussaint, un de ses anciens élèves du Service de santé militaire, la cravate de commandeur de la Légion d'honneur [32].

## Expertise Sur Le Paludisme En Macédoine Grecque

### Le front de Salonique

En 1914, Laveran, presque septuagénaire, reprend du service pour faire partie de la Commission supérieure consultative d'hygiène et d’épidémiologie militaires, dont il restera membre jusqu’à la fin de ses jours à Paris le 18 mai 1922 [13]. Son *Traité du paludisme*, dont la dernière édition date de 1907, sert de référence pendant la Grande Guerre. Le front de Salonique en Macédoine grecque mis en place fin 1915 va être l'occasion pour Laveran d'apporter son expertise. Dès janvier 1916, Laveran et les frères Edmond et Étienne Sergent, qui luttent avec succès contre le paludisme en Algérie depuis 1900, vont avertir les autorités militaires sur le grave danger que fera courir le paludisme aux troupes de l'Armée française d'Orient (AFO) dans la plaine marécageuse du fleuve Vardar à Salonique – « l'hydre du Vardar » comme le surnommaient les deux frères [33].

### Prophylaxie du paludisme pour l'Armée française d'Orient

Laveran rédige rapidement un document repris en Instruction sur « la prophylaxie du paludisme dans l'Armée d'Orient », qui sera publié secondairement en 1917 dans le bulletin de la Société de pathologie exotique (SPE) [27]. Société qu'il avait créée en 1908 et qu'il présidera pendant 12 ans [9, 45]. Les stratégies de contrôle recommandées par Laveran, qui restent pour l'essentiel, hormis les insecticides qui n'existaient pas à cette époque et de nouveaux antipaludiques, celles encore mises en œuvre en 2022 dans les armées françaises [34], sont les suivantes:

- assainissement de l'environnement;
- choix des implantations des cantonnements loin des « populations endémisées »;
- lutte antivectorielle avec usage de moustiquaires et lutte contre les larves;
- quinine préventive obligatoire pour les troupes avec surveillance de la prise;
- quinine à fortes doses pour les malades;
- quinine également pour les réfugiés vivant au sein du camp militaire de Salonique;
- conférences par des médecins aux officiers puis causeries de ceux-ci à leurs hommes.

### Catastrophe sanitaire en 1916

Malgré ces mesures, la catastrophe annoncée se produit dans l'AFO entre juin et décembre 1916 avec 30 000 cas recensés, 60 000 aux dires des médecins, 630 décès touchant principalement les troupes du Train, du Génie et des Territoriaux, 20 000 rapatriés en métropole et seulement 20 000 hommes en ligne en fin d'année. Le commandant en chef des armées alliées, Maurice Sarrail, avertissait le ministre de la Guerre par cette phrase devenue célèbre: « Mon armée est immobilisée dans les hôpitaux [33]. »

### Nouveau plan de campagne antipaludique en 1917

En janvier 1917 le sous-secrétaire d’État Justin Godart, au nom du ministre de la Guerre Hubert Lyautey futur maréchal de France, fait appel aux frères Sergent pour élaborer un « Plan de campagne antipaludique pour l'Armée d'Orient » sur le modèle de leur programme algérien et pour le mettre en œuvre sur le terrain [33, 41]. Plusieurs des stratégies de ce plan sont identiques à celles recommandées par Laveran. Certaines sont plus novatrices et adaptées à une armée en campagne comme:

- la mission antipaludique autonome avec ses propres moyens humains et matériels;
- la cartographie du risque palustre sur 8 secteurs d'activité;
- le contrôle urinaire de la prise de quinine à dose préventive ou curative;
- et la propagande moderne, notamment la diffusion d'un jeu de 10 cartes postales rappelant les règles de prévention du paludisme, créées avec l'aide de René Legroux de l'Institut Pasteur et illustrées par Albert Guillaume [33].

### Contribution à la victoire finale en 1918

**Figure 1:**
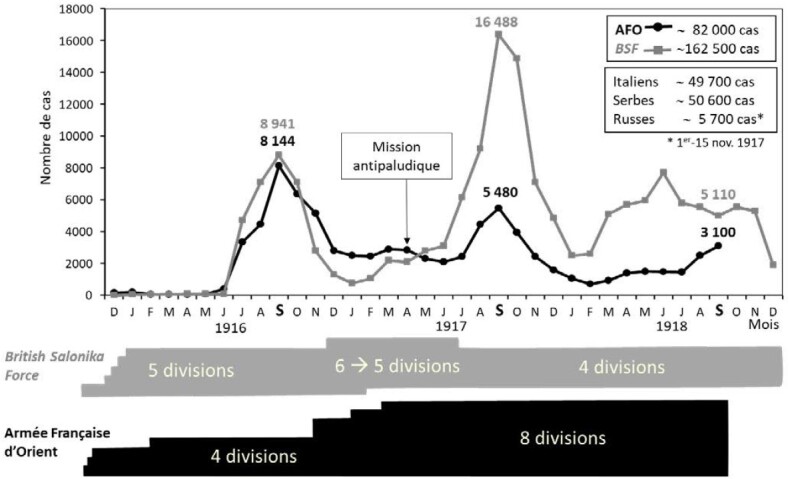
Comparaison du nombre de cas de paludisme de l'Armée Ffrançaise d'Orient (AFO) et de la British Salonika Force (BSF) de décembre 1915 à décembre 1918 sur le front de Salonique en Macédoine grecque [Migliani, données personnelles non publiées] Comparison of the number of malaria cases of the French Army of the East and the British Salonika Force from December 1915 to December 1918 on the Salonika front in Greek Macedonia [Migliani, unpublished personal data]

Dès l’été 1917, on observe une baisse significative du nombre de cas qui va se poursuivre jusqu'en septembre 1918 (Fig. 1) [33]. C'est le moment choisi par Louis Franchet d'Esperrey, commandant en chef de l'AFO et futur maréchal de France, pour déclencher une offensive victorieuse contre l'armée bulgare. Cette victoire décisive contribuera à l'Armistice du 11 novembre [33].

À l'arrière, Laveran favorise tout au long du conflit la valorisation des travaux des médecins de la mission antipaludique en les publiant dans le bulletin de la SPE, notamment la cartographie du risque palustre [33].

## Conclusion

Tant ayant déjà été dit sur la vie de labeur et l’œuvre d'Alphonse Laveran le médecin militaire, nous conclurons par une anaphore:

« Un fils de » dont le père Louis-Théodore, illustre médecin militaire, professeur, titulaire de Chaire et directeur du Val-de-Grâce, fut un modèle;

« Un fils de » qui apprivoise, dès ses études médicales, le microscope qui l'aidera dans sa future carrière de chercheur;

« Un fils de » qui affronte, jeune médecin militaire, les feux de la guerre et de la révolution;

« Un fils de » dont le père est mort en août 1879, quasiment un an avant la découverte de l'hématozoaire du paludisme qui conduira Alphonse à la postérité [14];

« Un fils de » qui fait depuis 115 ans, depuis son prix Nobel, la gloire du Service de santé des armées et de l'Institut Pasteur;

« Un fils de » d'une grande modestie, qui souhaite dans ses dernières volontés « ni discours, ni délégations officielles, ni fleurs, ni couronnes » et dont l’épitaphe au cimetière parisien du Montparnasse, près de la tombe de François Maillot, porte ces seuls mots: « Membre de l'Institut et de l'Académie de médecine » [15, 28, 44].

## Remerciements

L'auteur adresse ses sincères remerciements à Marc Morillon.

## Liens D'intérêts

L'auteur ne déclare aucun lien d'intérêt.
